# Utility of Photodynamic Therapy in Dentistry: Current Concepts

**DOI:** 10.3390/dj8020043

**Published:** 2020-05-07

**Authors:** Anette Stájer, Szilvia Kajári, Márió Gajdács, Aima Musah-Eroje, Zoltán Baráth

**Affiliations:** 1 Department of Periodontology, Faculty of Dentistry, University of Szeged, Tiszta Lajos körút 62-64, 6720 Szeged, Hungary; szilvia.kajari@gmail.com; 2 Department of Pharmacodynamics and Biopharmacy, Faculty of Pharmacy, University of Szeged, Eötvös utca 6, 6720 Szeged, Hungary; gajdacs.mario@med.u-szeged.hu; 3 Department of Prosthodontics, Faculty of Dentistry, University of Szeged, Tiszta Lajos körút 62-64, 6720 Szeged, Hungary; aima1993@hotmail.co.uk (A.M.-E.); barzol34@gmail.com (Z.B.)

**Keywords:** photodynamic therapy, photosensitizer, aminolevulinic acid, reactive oxygen species, oral medicine, endodontics, periodontology, implantology, caries

## Abstract

The significant growth in scientific and technological advancements within the field of dentistry has resulted in a wide range of novel treatment modalities for dentists to use. Photodynamic therapy (PDT) is an emerging, non-invasive treatment method, involving photosensitizers, light of a specific wavelength and the generation of singlet oxygen and reactive oxygen species (ROS) to eliminate unwanted eukaryotic cells (e.g., malignancies in the oral cavity) or pathogenic microorganisms. The aim of this review article is to summarize the history, general concepts, advantages and disadvantages of PDT and to provide examples for current indications of PDT in various subspecialties of dentistry (oral and maxillofacial surgery, oral medicine, endodontics, preventive dentistry, periodontology and implantology), in addition to presenting some images from our own experiences about the clinical success with PDT.

## 1. Introduction, History of Photodynamic Therapy

Over the past century, the significant growth in scientific and technological advancements within the field of dentistry has resulted in a wide range of novel treatment modalities that are available for dentists to use; these advancements have revolutionized the dental care of patients in the 21st century [1,2,3]. One such advancement is the use of photodynamic therapy (PDT), which is an emerging, non-invasive treatment method, involving chemical agents as photosensitizers (PS), light of a specific wavelength and the generation of singlet oxygen (^1^O_2_) and reactive oxygen species (ROS) in the presence of endogenous molecular oxygen to eliminate unwanted eukaryotic cells (e.g., malignancies in the oral cavity) or pathogenic microorganisms (e.g., in bacterial, fungal or parasitic infections) [4,5]. The utility of PDT in medicine is a relatively recent discovery, however, the earliest description of its theoretical basis and application can be dated back all the way to the ancient Egyptians [6,7]. According to their beliefs, the Sun alone possessed healing powers against certain skin conditions, such as vitiligo, psoriasis and skin cancer [6,8]. In addition, the pivotal “Ebers Papyrus” (being one of the oldest and most important Egyptian ancient medical texts, dating back to about 3000 B.C.) describes the use of plants, such as parsnips (*Pastinaca sativa*) and parsley (*Petroselinum crispum*) to make powders, which were placed on depigmented lesions of the skin [9,10]. Upon exposure to sunlight, this resulted in skin pigmentation, an effect that was similar to a sunburn. Whilst the ancient Egyptians were the pioneers of this method, their theoretical understanding (i.e., beliefs) of the phenomenon was later proved to be incorrect, based on novel scientific advancements [9,10]. In addition, the ancient Greeks were also among the first people to utilize phototherapy in the history of mankind. Today, this practice of using sunlight (or light in general) as a therapeutic agent is known as phototherapy or heliotherapy [11,12]. The term “heliotherapy” is of Greek origin, and its creation is attributed to Hippocrates (460–370 B.C.), the “Father of Medical Science”. After travelling to Egypt, he recommended sunlight for the restoration of health. Civilizations in India and China have also described the healing properties of sunlight for various pathologies [7,11,12].

In the Western world, the basics of modern phototherapy were first established in the 1890s, by the Danish scientist Niels Finsen, who worked extensively with light sources ranging from small active rays to ultraviolet radiation; his research enabled others to later use these light sources as a therapeutic modality against lupus vulgaris and smallpox [13,14]. For his innovative contribution to medical science (highlighting the significance of this topic), he was awarded a Nobel Prize in 1903 [15]. Another discovery of paramount importance was by the German medical student Oscar Raab and his supervisor Prof. Hermann von Tappeiner in 1900; during their search for novel anti-malarial drugs, the medical student noticed that paramecia (a type of aquatic microorganism, which was used as a model organism at that time) incubated with acridine orange (AO) dye died at a faster rate after a thunderstorm, compared with when there was no storm [16]. This experiment provided similar results when the AO-treated paramecia were exposed to sunlight from the adjacent window, compared with when incubated in a dark room. This serendipitous discovery led von Tappeiner to postulate that the light may have played a role in accelerating the chemical–biological reaction. This phenomenon was termed “photodynamiche” (german for photodynamic effect), and his theory was that oxygen was required for the photosensitization process to occur [17]. In a book published by von Tappeiner in 1907, the results of his clinical experiments (in collaboration with the German dermatologist Albert Jesionek) were summarized: they used the xanthene dye eosin in conjunction with illumination to treat basal cell carcinoma of the skin, condyloma acuminata of the female genitalia and lupus vulgaris, resulting in favorable outcomes [18]. This was the first, real clinical use of PDT to treat a disease, in addition to contributing critically to the emergence of PDT [16,17].

PDT was revolutionized (and the complete understanding of the mechanism of action was attained) after the boom in the (bio)chemistry field and of porphyrin compounds. In 1913, the Austrian physician Fredrich Meyer-Betz experimented on himself, with an IV injection of 200 mg hematoporphyrin (a derivative of protoporphyrin IX, where the two vinyl groups have been hydrated) [19,20]. Upon light exposure, he noted the development of extreme pain and swelling, which was confined to the areas exposed to light; this area remained photosensitive for several months after the incident. He concluded that hematoporphyrin was a PS agent. Further research led by Schwartz et al. in 1955, also recognized that a hematoporphyrin derivative was a more efficient PS, and in comparison, this molecule targeted cancerous cells more effectively and gave better overall results [21]. Their results highlighted that hematoporphyrin accumulates in cancerous tissues, which further sped up the development of novel porphyrin-based photosensitizers and their application in the treatment of malignant disorders [21]. The additional surge of interest towards PDT began after the studies on various hematoporphyrin derivatives by Lipson and Schwartz at the Mayo Clinic in the 1960s, which was further accelerated by pioneering studies in both basic science and clinical applications by Dougherty et al. [22]. Their research group (together with the topic of PDT) has gained recognition after they conducted clinical PDT trials on a global scale; in addition, they have established (in 1986) the International Photodynamic Association and expanded it to almost every country around the world [22,23]. As a result, PDT was approved by the Worldwide Food and Drug Administration in 1999 to treat precancerous skin lesions of the face or scalp and has extensively been used to treat cancers and certain other diseases. In parallel, Photofrin^®^ (partially purified version of hematoporphyrin) received approval for the management of several malignant disorders in the majority of developed countries [24]. To date, Photofrin^®^ is the most extensively studied and clinically used photosensitizer. PDT has been proposed to be useful in almost all facets and specialties of medicine, and the possible applications keep expanding every day [25]; however, there is a limited amount of publications on the relevance of PDT in dentistry. Currently, the main indication of phototherapy is to treat infants born with neonatal jaundice—a treatment that was developed in the United Kingdom some 50 years ago [26].

The aim of this review article is to summarize the history, general concepts, advantages and disadvantages of PDT and to provide examples for the current indications of PDT in various subspecialties of dentistry, in addition to presenting some images from our own experiences about the clinical success with PDT.

## 2. The Theoretical Basis of PDT

PDT is a therapeutic alternative, combining photophysical and photochemical processes, resulting in biological effects [27]. The process includes the excitation of PS with light (a physical process), which is followed by photochemical reactions of the excited PS with various cellular substrates and molecular oxygen, eventually leading to cell death. Interestingly, each element of PDT is non-toxic individually (generally, although some PSs may exert toxic adverse events in larger doses), however, when the photosensitive material is coupled with light of a specific wavelength, the resulting chemical reaction results in the formation of toxic species, causing cell death by various molecular mechanisms [28]. In the following section, the main components of PDT and the mechanism of action for these treatments are described.

### 2.1. Photosensitizers (PS)

**Figure 1:**
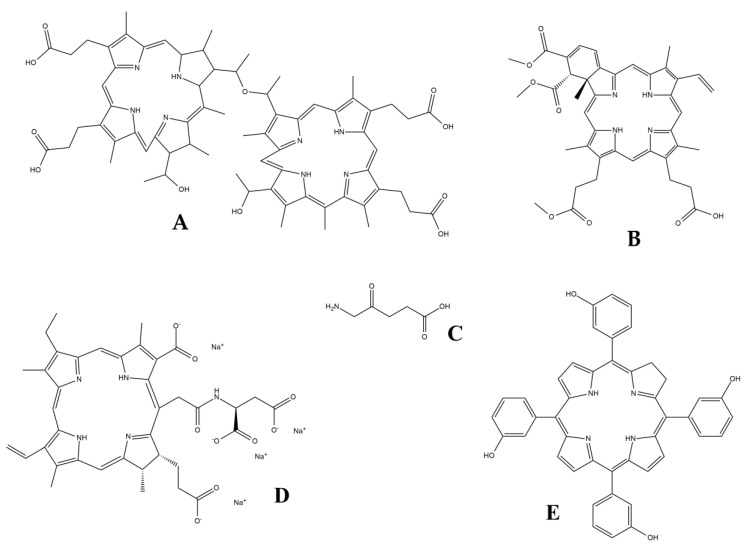
Examples of clinically relevant photosensitizer (PS) compounds. (**A**) Photofrin^®^ (hematoporphyrin or dihematoporphyrin ether); (**B**) Visudyne^®^ (verteporfin); (**C**) 5-aminolevulinic acid (ALA); (**D**) talaporfin sodium (LS11); and (**E**) Foscan^®^ (temoporfin).

PS agents are special compounds used in PDT. Depending on the type of agent used, the PS is either intravenously injected into the bloodstream (where it will travel to its target area), ingested orally or it is applied topically to the location that requires treatment [29]. In order to take effect, photosensitizers require activation by a well-defined wavelength of light, which will initiate the mechanism needed to target and eradicate unhealthy tissue [30]. Regarding their chemical structure, PSs are usually macrocyclic compounds with a heterocyclic ring structure that is similar to chlorophyll or heme, although other PSs have also been described, e.g., some pharmaceutical compounds possessing photosensitizing effects (e.g., phenothiazines, sulfonamides, psorales, hypericin) (Figure 1) [29,30]. They can be classified based on their chemical structure but are more commonly grouped into three broad families based on their clinical characteristics [31].

First generation PSs have been available since the 1970s and the early 1980s. Most of these compounds are cyclic tetrapyrroles, comprising substituted derivatives of porphyrin, chlorin and bacteriochlorin, while clinically-relevant compounds most frequently are structural derivatives of hematoporphyrin [29,30]. First generation agents have many drawbacks, e.g., high aggregation tendency, lack of specificity, low solubility in physiological liquids and cutaneous phototoxicity. Thus, most of the first generation PSs are unsuitable (by current standards) for use in PDT, but they provided a source for the synthesis of new PSs that have complied with modern requirements from pharmaceutical compounds [32]. Hematoporphyrin is commercially known as Photofrin^®^, which is the most extensively studied and clinically used photosensitizer to date. This PS agent was approved for the management of lung, bladder, esophageal and early stage cervical cancers in the 1950s, while its significance as a PS agent was discovered years later [33,34]. Additionally, hypericin, eosin, methylene blue and rose bengal have also been previously employed as PS agents; nowadays, they are used in different indications [35].

Second generation PSs (e.g., verteporfin, talaporfin, temoporfin) were developed in the late 1980s, as an attempt to improve the efficacy of first generation agents, in addition to gaining better pharmacokinetic properties and a lower toxicity [29,30]. In addition, these PSs have a near infrared absorption and a high ^1^O_2_ yield compared with the first generation compounds [36]. These molecules include core or structurally modified or substituted porphyrins, bacteriochlorins, chlorins, phthalocyanines or other macrocyclic compounds [37]. In addition to the abovementioned tetrapyrrolic compounds, fullerenes (allotrope structure of carbon, composed of 60 carbons arranged in soccer ball shape) are another class of novel second generation PSs [38]. Chemically, the presence of condensed aromatic rings in these molecules leads to an extended p-conjugation that is a desirable property for PS molecules; nevertheless, the functionalization of these compounds is necessary for them to be soluble in biological solvents [37]. The most commonly used and well-known are 5-aminolevulinic acid (ALA; acting as a pro-drug) and a structurally modified version of hematoporphyrin (such as benzoporphyrin derivatives) [29,30,39]. ALA is a so-called intrinsic photosensitizer, that is converted in situ to protoporphyrin IX; the introduction of exogenous ALA in vivo inhibits the first step of porphyrin synthesis, resulting in the accumulation of protoporphyrin IX in the tissue [29,30,39]. Topical ALA and its ester derivatives have been approved by the FDA and used to treat many diseases, like pre-cancer conditions, basal and squamous cell carcinoma of the skin, Bowen’s disease, and actinic (solar) keratoses and gastrointestinal cancers [40].

Third generation PSs are the most recently developed compounds of medical importance; the derivatives of the second generation PS compounds possess various functional groups to which conjugation is possible by multifarious synthetic strategies, therefore this may lead to several advantages in their use [29,30]. As a general rule, these are second generation PS compounds that are usually conjugated with some biological molecules or they have built in “photo-quenching” properties, i.e., these photosensitive materials only become activated at their specific target site (e.g., protein, receptor) [41]. Possible carrier molecules for the former group includes monoclonal antibodies, non-antibody-based protein carriers, monosaccharides, polymers, polymeric nanoparticles (NPs) or liposomes; while cellular markers for the latter group include tumor surface markers (e.g., epidermal growth factor receptor), receptors (e.g., low-density lipoprotein (LDL) receptors, transferrin receptors, folic acid receptors, integrin receptors) and transporters (e.g., glucose transporters) [29,30,41]. The conjugation of fullerenes with polyethylene glycol (PEG) increases their tumor localization and increases their solubility in water-based solvents and in vivo biological conditions [38].

The requirements for an optimal photosensitizer are the following: commercial availability in its pure chemical form, cost-effectiveness, ease of administration, long wavelength absorbing-capacity, low dark toxicity but strong photocytotoxicity, good selectivity towards target cells and rapid elimination [27]. Although there is currently no PS which adheres to all the above-mentioned criteria, this list provides a general guideline for the development of novel agents [25]. Currently, there are only a few PSs that have received official approval for clinical use around the world, thus it is imperative to carry out more research in this field to find additional compounds for treatment regimens [29,30,37,41]. The use of PSs may be activated by daylight, leading to first or second degree burns to the skin, which may discourage both clinicians and patients from using these agents [42]. It is suggested that patients can get around this by avoiding direct sunlight for several hours, until the drug is fully eliminated from the body, however, this is not always possible. There is a need for more research and investment to find alternative PSs that work with the same or an improved efficacy, but with shorter half-lives and rapid elimination from the body [29,30,37,41]. In addition, the development of novel PSs should address issues with mutagenicity selectivity and the more precise targeting of PSs, dependable activation by an appropriate wavelength of light (both of which were the main objectives during the development of third generation PSs) and options for pain-free outpatient therapy [29,30,37,41,43,44]. The possible development of photosensitizers with longer activation wavelengths will also allow for deeper tissue penetration.

### 2.2. Light Source

The clinical approach of PDT largely depends on the choice of the appropriate light (at a specific wavelength), light delivery (to activate the PS) and a sufficient PS concentration in the presence of oxygen at the target tissue [45]. All the main factors of PDT light delivery have an important role to support successful treatments, which results in its excellent therapeutic effects; nonetheless, treatment specifications (light dose, dose rates, resulting tissue destruction) are characteristic to each tissue type, which must be considered before therapy [45,46]. The light wavelength is directly proportionate to the tissue penetration, with light (i.e., red light in the visible wavelengths) that is transmitted easily through tissues being optimal for use in PDT [47,48]. The range of light to be used in PDT ranges between 600–900 nm; below 600 nm is unsuitable as endogenous molecules (e.g., heme) would predominantly capture most incoming photons. In contrast, the energy content of the photons above 900 nm is not enough to generate ^1^O_2_ species [49]. In clinical practice, most PSs are activated by red light (with a wavelength ranging between 630–700 nm), which corresponds to a penetration depth range of 0.5–1.5 cm. This limits the depth of tissue death and defines the therapeutic effect [45,46].

The delivery of light for PDT is characterized by the indication of PDT, the size, shape and anatomical location of the lesion [45,46]. Additionally, the intensity of the produced light should be uniform, to allow for dose calculations during treatment. In the beginnings of PDT, so-called non-coherent light sources (phosphor-coated sodium lamps, tungsten filament, quartz halogen, xenon arc and metal halide lamps) were used; the advantage of these lamps is that they can produce spectra of wavelengths to accommodate various photosensitizers (additionally, if combined with optical filers, they can produce selective wavelengths), they are safe, inexpensive, easy to use and they can cover large areas [45,46,50,51]. However, as they release a significant amount of heat, have a low light-intensity and poor dose control, they are not extensively used [45,46]. Lasers (including gold or copper vapor-pumped dye lasers, argon-pumped dye lasers and potassium titanyl phosphate (KTP)- or neodymium/yttrium aluminum garnet (Nd/YAG)-pumped dye lasers) also represent a viable light source, however, laser-based light sources are very complex to handle, labor-intensive and expensive [34,45,46,52]. Light-emitting diode (LED)-based laser systems are an emerging light source for PDT because they are cost-effective, portable and easy to handle overall [45,46,50]. Another advantage of LED-based systems is that the tip of these emitting fibers may be made in various shapes and uniform diffusion may be achieved with the use of a light applicator (or so-called diffuser) [53].

### 2.3. Role of Oxygen and Oxygen Species, Molecular Mechanisms of Action

**Figure 2:**
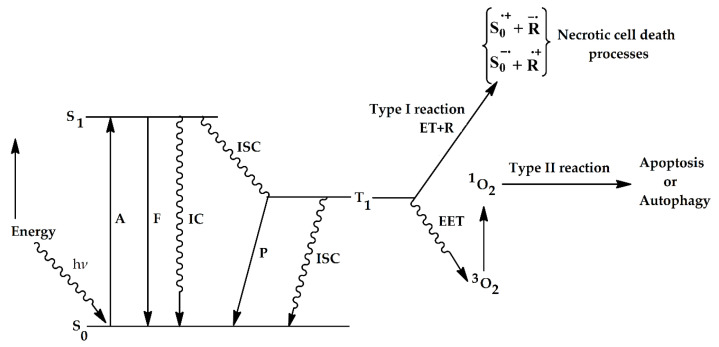
A diagram, illustrating the various changes in energy levels, which provide the physical and molecular basis for photodynamic therapy (PDT) (adapted from [25]). ^1^O_2_: singlet oxygen, ^3^O_2_: triplet oxygen, A = absorption of photons, F = fluorescence (emission), P = phosphorescence S = singlet state, T = triplet state, IC = internal conversion, ISC = intersystem crossing, EET = energy transfer through excitation, and ET = transfer of electrons, R = substrate.

Oxygen is the last component needed for the photodynamic reaction to take place [24,48]. Several studies have shown that PDT efficacy is an oxygen-dependent process [54]. This oxygen dependence is generally believed to be required for the generation of ^1^O_2_ and ROS, which are believed to be responsible for most photodynamic processes in biological systems [55]. The photodynamic process begins with the excitation of a photosensitizer (PS) using a light source, which leads to subsequent photochemical reactions of the excited PS with the cellular substrates or molecular oxygen that ultimately result in cell death [25]. In its ground state, the photosensitizer has two electrons (e-) in opposite spins in the low energy molecular orbitals [10,56]. When light is absorbed, one of these e- is excited to a higher energy molecular orbital, without changing its spin; this is called the singlet excited state [57]. In order to return to the ground state, the excited PS will do one of two things: it will a.) radiate energy in the form of light (fluorescence) or b.) result in non-radiative decay through the release of heat energy during a process called internal conversion (IC) [58,59]. If the electrons in a molecule from a singlet ground state become excited to a higher energy level (through the radiation of absorption), they may either form an excited ^1^O_2_ (where the pair of electrons on the same energy level have opposite spins) or a ^3^O_2_ (the excited electron is in parallel, i.e., they have the same spin as the ground state electron). The radiationless process of transition between two electronic states of spin multiplicity (in this case, between the ^1^O_2_ and ^3^O_2_ state) is termed intersystem crossing (ISC) [25,58,59]. The ^3^O_2_ state PS will also dissipate its energy by radiative (phosphorescence) or by nonradiative processes (releasing energy in the form of heat) in order to come back to the ground state [58,59]. During PDT, the excited ^3^O_2_ state will either directly react with cellular substrates by the transfer of electrons and eventually produce oxygenated products (Type I reaction) or it will transfer energy to molecular oxygen, leading to the generation of highly reactive ^1^O_2_ species (Type II reaction) within the biological environment (summarized in Figure 2) [25,60,61,62]. The ^1^O_2_ oxygen is the most damaging species as it reacts with biological molecules, e.g., unsaturated lipids and amino acids of proteins (tryptophan, histidine and methionine) [25,60,61]. A singlet excited state PS is unable to react with cellular substrates, as its lifetime is very short (ranging from nano to pico seconds), whereas a triplet state PS, that has a significantly longer lifetime (ranging from micro to milli seconds) can efficiently carry out these reactions [25,60,61,63]. The biological outcome of PDT on a cellular level is dependent of the reaction type that occurs; Type I reactions usually result in necrosis, a sudden, rapid cell death mechanism that effects a large numbers of cells simultaneously, without sparing the surrounding cells; this “collateral damage” is mediated by the release of cellular materials and cytokines into the surrounding extracellular environment [25,60,61,64]. Generally, higher doses of a PS and high fluorescence rates lead to a more extensive destruction of the cell membrane, leading to necrosis [65]. The formation of ROS is the main cause of necrosis during PDT. Type II reactions will result in either apoptosis or autophagy; apoptosis is a programmed cell death (PCD) mechanism, induced by a variety of intracellular signaling pathways, which is considered the primary mechanism of cell death in PDT [64]. Consequently, low doses of PDT-related damage most often lead to apoptosis, whereas high-level damage mainly leads to necrosis. During the early stages of apoptosis, morphological changes occur (the condensation of chromatin and nuclear/DNA fragmentation), while later on—depending on the apoptotic pathway initiated—the activation of the conserved biochemical cascade occurs [66,67,68]; in the late stages of the intrinsic apoptotic pathway, this results in the activation of the apoptosome and caspase-9, while in the extrinsic pathway, the processes are mediated by caspases-8 and -10. Consequently, the activation of both pathways leads to the activation of endonucleases and caspases-3 and -7, and subsequent cell death [66,67,68]. In contrast, autophagy is a regulated biochemical mechanism, where the apoptotic “machinery” is absent; during this process, unnecessary or dysfunctional cellular components are removed by a self-digesting mechanism, using lysosomes, where lysosomal enzymes degrade and recycle these components [62,66]. It must be noted that the type of PS, the extent of the generated ROS and the level of photo-induced damage all influence the extent of autophagy, which may be both cytoprotective and cytotoxic [66].

## 3. Application of PDT in the Various Fields of Dentistry

### 3.1. PDT in Oral and Maxillofacial Surgery, Oral Medicine and Oral Surgery

PDT has proved itself to be a promising tool in the treatment of pre-malignant and malignant lesions of the head and neck region, including the oral cavity [33,46]; while the use of PDT in the diagnosis of these lesions in the oral cavity is a relatively new, it is an important advancement in dentistry. In recent practice, the topical application of the photosensitizer ALA is used as a diagnostic tool for oral lesions, in a procedure known as ALA-based photodynamic diagnosis [69]. ALA is topically applied to the suspected lesion, where it accumulates and increases the tissue fluoresce of the lesion when it is illuminated. The measurable difference between the fluorescence levels of normal and pre-malignant tissues allows for the distinction between malignant and non-malignant lesions [27,69]. Evidence of its utility is depicted in a study by Sharwani et al. [70], where patients with suspected oral leukoplakia underwent an ALA-based photodynamic diagnosis. Afterwards, a surgical biopsy was taken from the same examination site. The results of the fluorescence spectroscopy were then compared with histopathology and showed that dysplastic lesions have a higher fluorescence than benign oral lesions without changes in the green autofluorescence, further verifying the validity of an ALA-based photodynamic diagnosis [69]. However, it is also important to note that ALA has a tissue penetration depth of 1–1.5 mm, so this method is only applicable for superficial lesions [39,71]. This explains the poor registration of some cytological and biochemical changes that occurred in some of the dysplastic tissues in the experiment. This diagnostic process is of paramount importance as oral leukoplakia and oral verrucous hyperplasia are some of the most common pre-malignant lesions that may transform into squamous cell carcinoma (SCC) or verrucous carcinoma (VC) of the mouth [72]. The detection of dysplasia in leukoplakia lesions increases the change for a subsequent malignancy to occur by 30% [72,73].

PDT is also known to be an effective treatment modality for pre-malignant lesions and early stage tumors of the head and neck region [56]. The advantage of PDT over conventional treatments is based on its minimal invasiveness and selective tumor destruction, with the preservation of healthy tissues [37,56]. This means that oral and facial prosthetics may be avoided since in many cases, PDT can be used over conventional surgical resection [4]. Here, the aesthetic advantage to the patient is obvious, as the use of facial prosthetics present many psychological challenges for the patient. The preservation of facial structures means patients’ quality of life will not be diminished as the ability of speech, eating and other activities will not be compromised by PDT [74]. Compared with conventional chemotherapy or radiotherapy as a treatment option, PDT poses the advantage that the number of sessions is not limited, and the side effects of PDT do not last as long and are not as severe [74,75]. Several PSs have the advantageous chemical property of concentrating in the histological site of malignancies, sparing healthy tissue. This has been demonstrated by studies that use the second generation photosensitizer temoporfin (m-THPC), commercially marketed as Foscan^®^ [34,35]. Dilkes et al. investigated the efficacy of Foscan^®^-mediated PDT within the years of 1996–2003. In one such experiment, 19 patients who had T1 and T2 grade lesions of the oral cavity and pharynx were treated with temoporfin [76]. Although 48% of the patients needed multiple sessions of PDT for the treatment to be effective, 90% of the patients showed a complete response; in addition, 10 out 19 patients remained disease free, within a follow-up period of 6–100 months [76,77]. The efficacy of temoporfin-mediated PDT in 25 patients with primary squamous cell carcinomas of the lip, over a period of 12 weeks, was also evaluated by Kubler et al.; 24 of the patients (96%) showed a complete remission after the treatment [78]. Of note, the remaining patients showed a partial response to the treatment, but were successfully re-treated with another session of m-THPC-mediated PDT, then subsequently showed a complete response at seven months after the re-treatment. Furthermore, the functional results were excellent in all the patients, without any signs of restricted mouth opening or impaired lip closure [78]. Although both experiments showed significant and promising results to argue for the use of PDT-mediated anticancer therapy, it is important to note that the lesions being experimented on are only pre-malignancies and early stage tumors of the head and neck; outcomes of this treatment strategy are not as good as in the treatment of advanced carcinomas with PDT [76,77,78]. This is probably due to a limited ability to adequately deliver laser light to the tumor because of the low penetration depth of Foscan^®^ [47]. Similarly, the utility of ALA was also demonstrated in pre-malignant lesions, however, due to the limited depth of the topical ALA, the use of this PS is restricted to superficial lesions [39].

**Figure 3:**
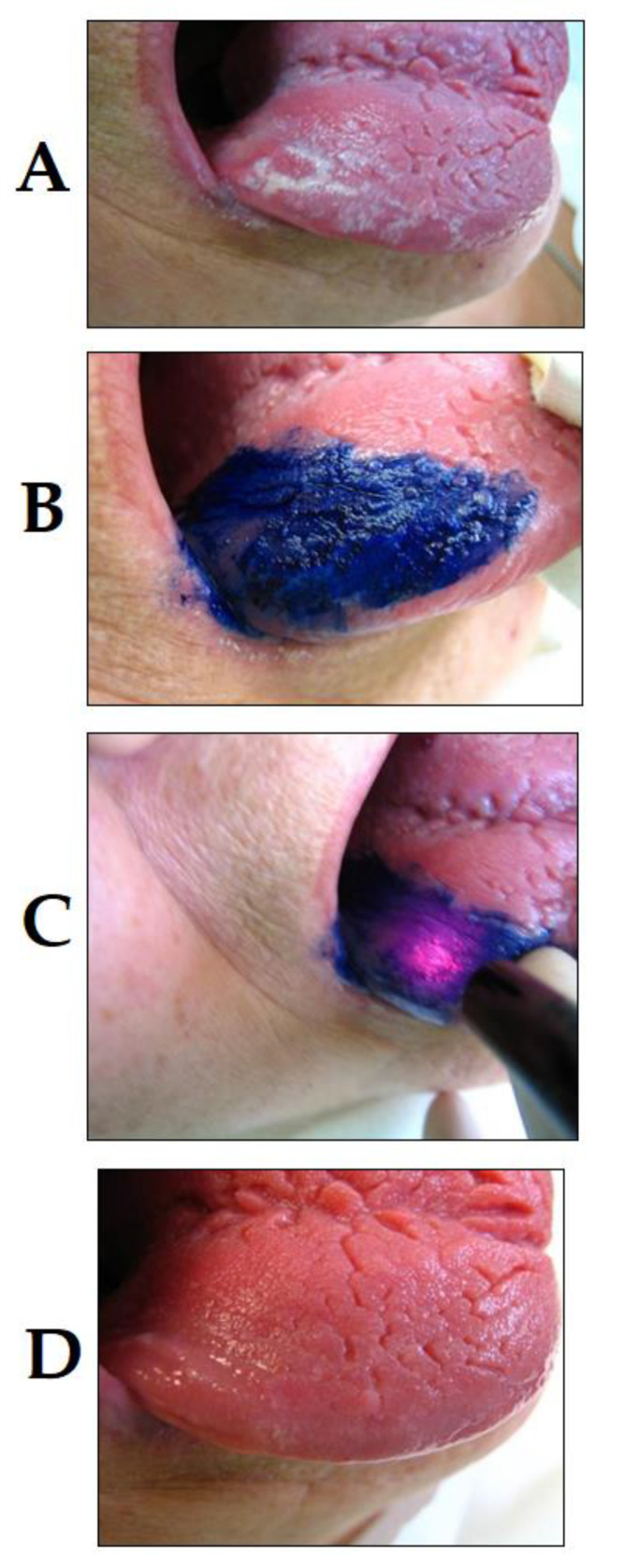
PDT of lingual candidiasis (photo courtesy of R. Szabó DMD, J. Arentz DMD and T. Nave DMD). (**A**) *C. albicans* infection of the tongue before PDT treatment; (**B**) application of PS compound; (**C**) irradiation; and (**D**) *C. albicans* infection of the tongue 36 h after PDT treatment, showing remission.

Among the various pathologies of the oral cavity, bacterial, viral and fungal lesions present an important factor for patient presentation in dental practices [79,80,81]. Although the use of PDT in infectious diseases of the oral cavity may seem uncommon, there is increasing interest toward this treatment modality in this field [82]. For example, oral candidiasis, a common fungal disease caused by *Candida* spp., frequently presents amongst patients wearing dentures, or patients with underlying immunosuppression, xerostomia, smoking, type-II diabetes, a hormonal imbalance or those undergoing hormone therapy [83]. Conventionally, oral candidiasis is treated using topical antifungal medications (solutions or creams), but these infections have the tendency to re-occur, especially in individuals who are presented with risk factors. Antimicrobial PDT has shown promising results in the treatment of oral candidiasis [84]. This was demonstrated in an in vitro study, which successfully used Photofrin^®^-mediated PDT to target various *Candida* species [85]. It is important to note that selectivity is an important factor, which needs to be taken into consideration during these treatments, as healthy human cells may also be destroyed using these agents, as well as the oral tissue infected with *Candida* spp. [86]. In oral candidiasis, a topical application may be applied by the selection of the affected areas only, and light can be applied to those regions only, making these infections treatable by PDT, without antifungal agents (presented in Figure 3). PDT has also been described to be used for the management of lesions in the oral cavity caused by the herpes simplex virus (or HSV), which are also commonly seen in clinical practices [87]. PDT, using methylene blue as a photosensitizer has been considered as an option in the treatment of herpes labialis; results have shown a decrease in the recurrence of vesicles and an increase in the comfort level of patients. The lesions in these patients healed more rapidly and significantly and no acute side effects of PDT were noted [88].

In oral surgery, there is relevance in the use of PDT in both the prevention and treatment of alveolar osteitis and post-extraction pain [89]. This was proven in 2004, when Neugebauer et al. used the photosensitizer HELBO Blue and a diode laser to successfully reduce the prevalence of alveolar osteitis [45]. Their study examined 100 patients who had at least one or multiple contralateral teeth extracted within the time interval of one week. One side (which was chosen at random) was treated with PDT, and the other side was treated conventionally in a standardized protocol. A recall appointment was given, and the extraction site was examined. The post-extraction pain was measured using an analogue pain scale of 0–100. The results showed that alveolar osteitis remained in 1 out of the 50 cases for the group that was treated with PDT, yet this was the case for 13 patients (out of 50) who were treated conventionally [45]. The pain assessment score for each group was scored after the extraction procedure and in the recall appointment a week later. After extraction, the scores for the group treated with PDT ranged 11.2 ± 9.8, while it was 19.0 ± 12.2 in the control group. The following week, the PDT group provided scores of 2.4 (±9.2), while the control group scored 13.1 (±25.2). This difference was significantly lower for the first and eighth days of post-surgery in the PDT group [45]. The researchers concluded that the significantly lower incidence of alveolar osteitis after PDT seems to be an emerging modality for the prevention of alveolar osteitis [89].

### 3.2. PDT in Endodontics

**Figure 4:**
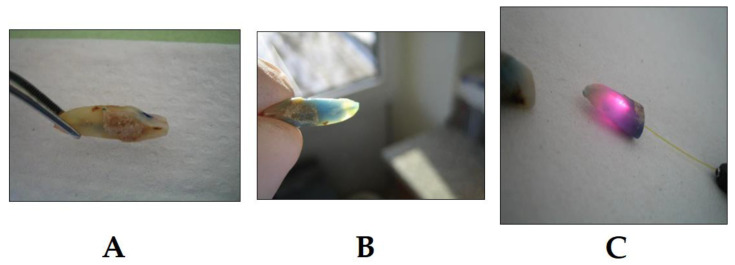
Example of PDT in endodontic treatment (photo courtesy of R. Szabó DMD, J. Arentz DMD and T. Nave DMD). (**A**) Extracted lower incisor after chemo-mechanical preparation and application of the PS into the canal; (**B**) penetration of the PS into the canal, 7 h after application, the PS already shows penetration into the apical region and lateral canals; and (**C**) treatment with laser light; the PDT light system is able to align the laser light in a way to allow for the activation of as many as possible PSs inside the canal to achieve the optimum effect. Following this step, root canals may be obturated.

In endodontics, it is essential to achieve and maintain sterility inside the root canal by the complete elimination of bacterial species colonizing it and causing infections [90]. This ultimately eliminates any chance of re-infection and allows for the healing of periapical tissues to occur [91]. Conventionally, this process is achieved by a mechanical treatment of the infected root canal, as well as the use of chemical irrigation agents. Recently, research has been conducted to support the use of PDT in conjunction with conventional chemo-mechanical preparations (Figure 4) [92]. The most recent evidence was shown by Okamoto et al.; after conventional root canal treatment was carried out on five anterior and deciduous teeth, PDT was conducted using the photosensitizer methylene blue and a 660 nm laser light [93]. The root canal was then irrigated with saline and a sealant was placed. Microbiological samples of the infected and disinfected root canal were taken, and the bacterial colonies were examined under a microscope. The results showed a bacterial reduction from 37.6% to a whopping 100%, further underlying the statement that PDT can be considered an alternative method to act as a support to root canal disinfection [93]. Compared with irrigation by the conventional sodium hypochlorite (which is considered to be the gold standard for root canal irrigation), PDT has shown very promising results. PDT has proven itself to be a very effective antibacterial agent, against both Gram-positive and Gram-negative endodontic bacteria [94], particularly *Enterococcus faecalis*, which has high levels of resistance to conventional chemo-mechanical irrigation systems; additionally, it is an extremely problematic species because of its resistance mechanisms to multiple antibiotics [95]. The photodynamic effect of methylene blue was investigated on *Actinomyces israelii*, *Fusobacterium nucleatum*, *Porphyromonas gingivalis* and *Prevotella intermedia*, all of which are common endodontic pathogens [96,97]. In this in vitro experiment, experimentally infected root canals of extracted teeth were treated. The results after a session of PDT using the methylene blue photosensitizer showed an 80% reduction in the colony forming units. The authors suggested PDT to be an effective method when used alongside a standard endodontic treatment, providing that the PDT parameters are at their optimum [90,96].

**Figure 5:**
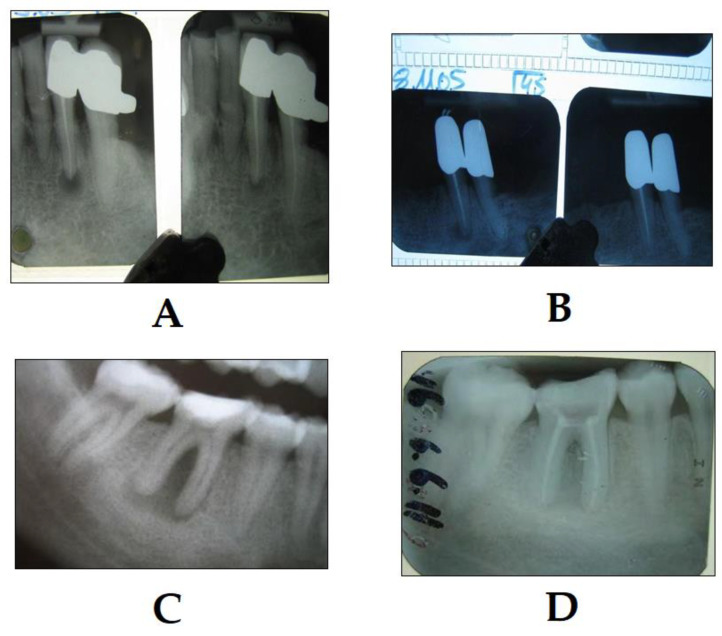
Success of PDT in the treatment of chronic periapical periodontitis (photo courtesy of R. Szabó DMD, J. Arentz DMD and T. Nave DMD). (**A**) The X-ray on the left shows the condition immediately after PDT, and the X-ray on the right shows the condition 4 months later; note the significant decrease in the size of the periapical lesion; (**B**) the X-ray on the left shows the condition immediately after PDT, and the X-ray on the right shows the condition 12 months later; note the significant decrease in the size of the periapical lesion. The same case is depicted in a lower molar tooth with chronic periodontitis, where (**C**) shows the condition immediately after the endodontic treatment and PDT, and (**D**) shows the condition after 3 months after the endodontic treatment, PDT therapy and root canal obturation; note the significant decrease in the size of the periapical lesion.

Chronic periapical periodontitis is a chronic inflammation of the periapical tissues, which usually occurs when bacteria from a necrotic tooth together with their toxins infect the periapical tissues surrounding the associated tooth [98]. Symptoms remain silent and the condition is usually discovered accidentally on a radiograph that shows a radiolucent lesion with sharp boarders. If left untreated, this small lesion may grow and become a radicular cyst [98]. Conventionally, this condition is treated with a two-visit root canal treatment that uses calcium hydroxide due to its antibacterial properties. In cases such as these, the endodontic therapy has a reduced success rate; commonly, these infections may persist, and retreatment may be required [94]. There is evidence to suggest that PDT may be beneficial as an adjunct to root canal treatment in the case of chronic periodontitis [25,99]. Garcez et al. enrolled 20 patients who suffered from chronic periapical periodontitis and had radiological symptoms [100]. A two-visit root canal treatment using PDT was performed, with polyethylene imine (PEI) as the photosensitizer and a fiber optic diode laser. Microbiological samples were taken on three occasions: firstly, after the access cavity was created, which highlighted the bacteria that were initially responsible for the infection; the second sample was taken after the chemo-mechanical preparation, and the final was taken after the PDT was performed. Following this, calcium hydroxide was placed in the root canal and the patient was recalled one week later for the same procedure to be repeated. The samples taken were examined microscopically and compared to each other. The results highlighted that including PDT as an adjunct in the treatment showed a significantly higher log reduction in the number of periodontal pathogens; overall, these results showed that the use of PDT in a two-visit root canal treatment enhanced the antibacterial effect of the treatment [100]. Figure 5 shows the representative cases from our practice: the X-rays were taken before and after PDT to measure the success of PDT in the treatment of chronic periapical periodontitis.

### 3.3. PDT in Preventive Dentistry

Dental caries is a chronic disease that usually begins on the oral surface of the teeth and spreads to deeper areas. It is the result of an imbalance between tooth demineralization and remineralization [101,102]. The demineralization is caused by acids released through the anaerobic carbohydrate metabolism of bacteria within the dental plaque, primarily by *Streptococcus mutans* [103,104]. Therefore, the formation of dental plaque is one of the hallmarks in the initial phases of caries development. It can be said that the elimination of the pathogenic microorganisms from the teeth by the removal of dental plaque is essential in the prevention and control of tooth decay [105,106]. This process can be achieved clinically by PDT-assisted plaque removal, a procedure also known as photodynamic antimicrobial chemotherapy (PACT) [107]. During this procedure, lasers of differing wavelengths are used as light sources with various PSs. Its use has been first described by Bevilacqua et al. [108], who used toluidine blue and an LED laser light, while the clinical utility of this method was investigated clinically by Wilson et al. [109,110]. Both in vitro and in vivo experiments yielded promising results [108,109,110]. It has been a well-known phenomenon that microorganisms may be eliminated by the combined used of dyes and light, however, the interest towards antimicrobial PDT was reduced by the discovery and clinical use of antibiotics [111]. However, this situation has changed drastically, due to the emergence and spread of multidrug resistant microorganisms (e.g., the ESKAPE group of bacteria [112]) and the recognition of the role of bacteria embedded in biofilms in the oral cavity (e.g., *P. gingivalis* in periodontitis) [113]. The advantage of this method is that it is presumed to be very unlikely that bacteria would develop resistance to the cytotoxic action of ^1^O_2_ or ROS (which have a direct effect on extracellular molecules), therefore, the use of PACT would not lead to further developments in resistance [114,115]. In addition, it has been proposed that polysaccharides present in the bacterial biofilm are also susceptible to this induced photo-damage; this (in addition to its multi-target antimicrobial mechanism) is an important advantage of PACT, compared with antibiotic therapy [107,114,115]. PACT should be considered as a potential alternative treatment, especially if the infection in question is localized in the oral cavity or the skin [116].

### 3.4. PDT in Periodontology

**Figure 6:**
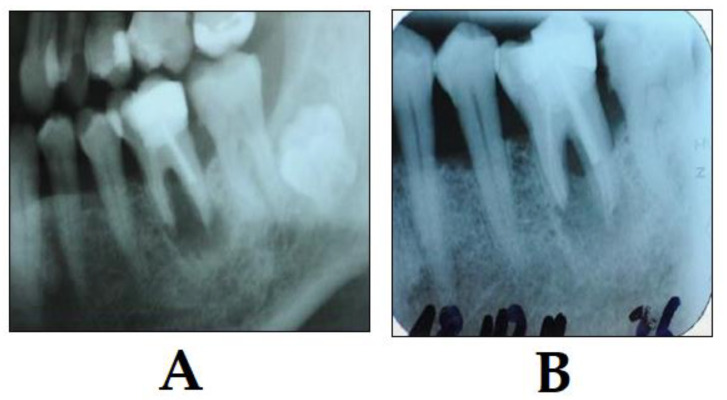
Example of PDT in periodontal therapy (photo courtesy of R. Szabó DMD, J. Arentz DMD and T. Nave DMD). (**A**) X-ray shows a perio-endo lesion of a tooth before PDT; and (**B**) X-ray shows the condition three months after PDT. Bone regeneration is visible showing clear signs of clinical improvement.

Successful periodontal therapy is based upon the ability to eliminate bacteria from the infected site. This is achieved by mechanical debridement of the bacteria through scaling root planning (SRP) and curettage, or through periodontal surgical methods; the measure is usually chosen depending on the level of damage to the periodontal tissues. As PDT is known to be an effective antibacterial technique, there is evidence to suggest its efficacy in the treatment of periodontal diseases (Figure 6) [107,114,115]. The idea that PDT can be used in the treatment of chronic periodontal disease has been proposed and investigated [117]. An in vitro study by Anderson et al. was carried out to compare the effectivity of PDT against conventional SRP in the non-surgical treatment of periodontitis [118]. Thirty-three patients with periodontitis—that ranged from moderate to advanced—were placed into three groups at random and treated with either PDT that used the methylene blue photosensitizer and a diode laser, SRP alone or a combination of PDT and SRP. The efficacy of each group was measured according to periodontal parameters that included bleeding on probing (BOP), probing pocket depth (PD) and clinical attachment level at 3-, 6- and 12-week intervals [118]. According to their results, the third group, who received a combination of PDT and SPR, showed the most promising clinical results. Braun et al. also demonstrated the positive effects of using PDT alongside subgingival scaling for the treatment of chronic periodontitis [119]. Twenty patients were enrolled in this study, who presented with chronic periodontitis; all teeth received subgingival SRP, followed by PDT treatment on two of the quadrants. The sulcus flow rate and BOP were examined at the initial baseline, at one week and then three months after the treatment. In addition, the relative clinical attachment level (RCAL), PD and gingival recession (GR) were evaluated at the baseline and three months after the treatment [119]. Their results showed an improvement in these factors by the three months in the group that received the additional PDT compared with the group treated with conventional methods. Some reports also suggest that the inclusion of PDT in the indication of aggressive periodontitis may also be relevant [120]. A split-mouth design study was conducted by De Oliveira et al., where 10 patients with aggressive periodontitis were treated by PDT that used phenothiazine as the PS and a 690 nm laser light in two quadrants; in contrast, the remaining two quadrants were treated by SRP including both hand and mechanical instruments [121]. The plaque index (PI), gingival index (GI), BOP, PD, GR and RCAL were measured at the baseline and three months after the treatment. Initially, the PI scores were 1.0 ± 0.5 in both groups; however, after the three-month evaluation, the plaque scores were reduced and remained low throughout the study. The results also showed that a pronounced change in the GI and BOP was shown in both groups after the three months [121].

### 3.5. PDT in Implantology

In dental interventions, peri-implantitis is a localized inflammatory process that affects the bone and tissue surrounding the dental implant. The occurrence of peri-implantitis is the result of bacterial contamination and colonization of the surface of the implant. When left untreated, peri-implantitis leads to implant failure as well as bone and tissue loss, significantly influencing the quality of life of the affected patients [122]. Conventionally, peri-implantitis is treated by decontamination using physical, chemical and mechanical means to remove as much bacteria as possible from the infected site [123]. However, the exact amount of bacterial and non-bacterial residue that must be removed from the implant surface to achieve stable and predictable clinical results post-treatment is unknown. Research into the application of PDT in the treatment of peri-implantitis was recently conducted by Dörtbudak et al., which aimed to assess the efficacy of PDT in peri-implantitis against *P. gingivalis*, *P. intermedia* and *Aggregatibacter actinomycetemcomitans* [124]. They used the PS toluidine blue and irradiation with a diode laser on the implant surface of n = 15 patients that showed clinical and radiological signs of peri-implantitis. Microbiological samples were taken at the baseline, after the treatment with the toluidine blue PS and using a combined treatment of toluidine blue that was irradiated using a diode laser at 690 nm [124]. The cultures from the three groups were quantitatively examined for the abovementioned microorganisms. The results showed that treatment of peri-implantitis with a PS alone (i.e., without light sensitization) resulted in significant reductions in *P. intermedia* and *A. actinomycetemcomitans* compared with the original values, while this was not established for *P. gingivalis*. In contrast, the results from the full PDT treatment using both the photosensitizer and laser light together showed significantly higher reductions of bacterial loads (including *P. gingivalis*), however, it is important to note the complete elimination of bacteria was not achieved [124]. This led the authors to conclude that PDT yielded favorable results in the treatment of peri-implantitis. There is evidence to suggest that the use of PDT in combination with mechanical debridement around the infected implant surface provides a useful tool in the treatment of peri-implantitis and peri-implant mucositis [125].

**Figure 7:**
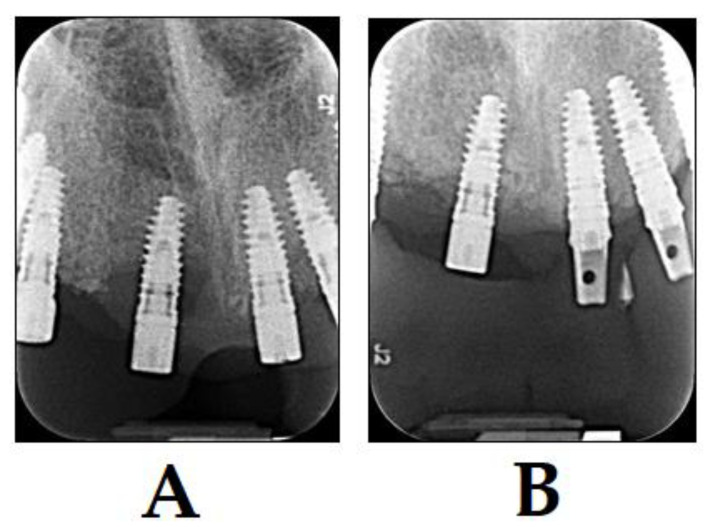
Treatment of patients with peri-implantitis with PDT and BIOOSS bone graft material (photo courtesy of R. Szabó DMD, J. Arentz DMD and T. Nave DMD). (**A**) X-ray of diagnosis of peri-implantitis before treatment; and (**B**) X-ray 3 months after treatment with PDT and BIOOSS bone graft material. Bone regeneration is visible showing a clear sign of healing.

**Figure 8:**
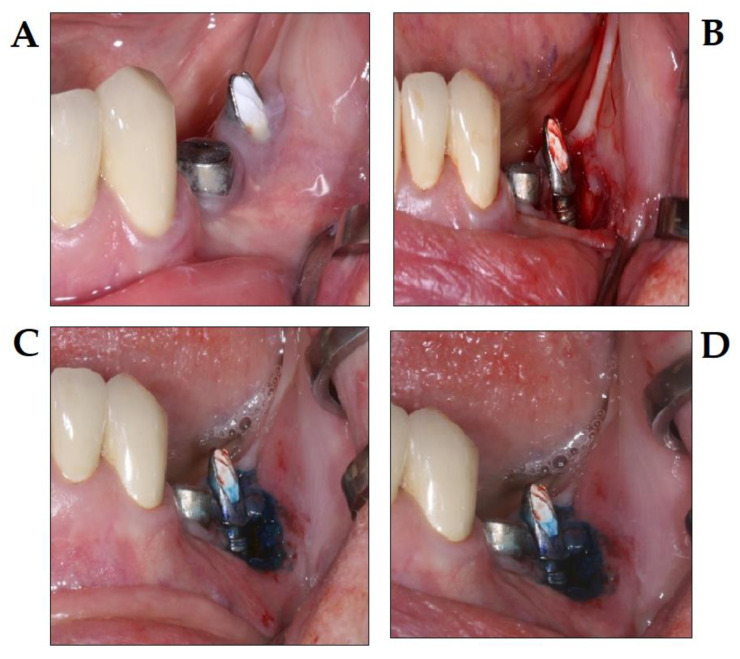
PDT in the treatment of periimplantitis, using the Photolase^®^ PDT system (photo courtesy of Z. Baráth DMD PhD). (**A**) Initial stage peri-implantitis; (**B**) surgical flap elevation; (**C**) application of photosensitizer after surgical debridement; and (**D**) conditions directly after the diode laser therapy.

However, these findings were recently challenged and contradicted by Esposito et al.; in their study, n = 80 patients were enrolled who showed clinical and radiological signs of peri-implantits. Forty of the patients were treated conventionally by non-surgical or surgical means, and the others were treated with PDT using the FotoSan system [126]. The study was carried out over the period of 12 months, followed by a follow-up evaluation. The criteria used to measure the success of the treatment were as follows: implant failures, recurrent peri-implantitis, implant complications, changes in peri-implant marginal bone level (RAD), PD changes and number of re-treatment sessions needed. In their report, one implant treated with PDT failed compared with the no failure of cases in the control group; in four patients, complications arose, and three of these patients were treated by PDT. The recurrence of peri-implantitis was observed in six patients (three from each group) and 29 implants were re-treated between 1–4 times in the PDT group vs. 33 re-treated implants 1–4 times in the control group [126]. Nevertheless, the peri-implant marginal bone levels remained stable, while PPD had significantly reduced in both groups at the 1-year mark, with no statistically significant differences between the groups. Based on these results, the authors concluded that the use of PDT in conjunction with conventional mechanical cleaning did not significantly improve the clinical outcomes [126]. Figure 7 and Figure 8 represent examples from our own practice about the use of PDT in periodontology.

## 4. Concluding Remarks

The currently available literature illustrates that the use of PDT within dentistry appears to be very promising, although the development of PDT is still in its “infant” stage. PDT offers a non-invasive treatment modality that has the potential to become applicable in all fields of dental medicine. Currently, the most frequent use of PDT clinical practice is in the treatment of cancers of the head and neck, showing various advantages compared with the conventional surgical resection methods. However, the use of PDT in head and neck oncology is limited by the inability of PSs to penetrate deeply into tissues, meaning it can only be used in the treatment of early and local neoplasms and not for advanced carcinomas. Further research into finding a photosensitizer that can penetrate deeper into tissues or one that can be used systemically must be undertaken to maximize its utility within this field. Photodynamic antimicrobial therapy is of great importance, particularly in an era when minimally invasive dentistry and prevention are at the forefront of the aims of dental medicine. In oral medicine, PDT has overcome problems associated with antibacterial, antiviral and antifungal resistance, and it can also be used against all microbes as many times as needed. Photofrin^®^ and some other PSs have received FDA approval for some 30 years now, and can be applied in the PDT of oral lesions, but many dentists are not aware of its application. Therefore, more advertising about the utility of PDT in oral medicine (and the same may be said about oral surgery) in the form of clinical trials and case studies are needed for dentists worldwide to become informed of the benefits of its use. The fact that PDT poses no threat of antibiotic resistance is what makes it so useful in endodontic applications. It must be noted that PDT will not replace conventional chemo-mechanical preparation methods, as it does not have the ability to mechanically remove infected tissue from the root canal like mechanical instruments, a substantive effect as high as chlorhexidine or a tissue dissolving ability like sodium hypochlorite; however, when used in conjunction with these conventional methods, results have been successful enough to merit its use. Although there is very limited information about PDT and its use in endodontics, PDT produces reliable results in the treatment of chronic periapical periodontitis. PDT offers an effective and professional way to remove plaque and therefore reduce the incidence of caries, a method of primary prevention. This method is highly effective against main caries-causing bacteria as well as other Gram-positive and Gram-negative strains. In order for the use of PDT as a preventative measure to become more popular, more clinical research showing its efficacy needs to be made available. Although the clinical evidence concerning the utility of PDT in periodontology is limited, PDT shows promising results, particularly in the treatment of chronic periodontitis. Research has concluded that PDT is at its highest efficacy when used as an adjunctive treatment to conventional debris meant methods that include SRC; PDT however, is not as effective in the treatment of aggressive periodontitis. Similar to periodontology, available research suggests that PDT in implantology is at its highest efficacy when used as an adjunct in the treatment of peri-implantitis, which is most likely due to its antibacterial characteristics. It must be noted however, that a lot of the practical cases in this field have been conducted on dogs and not humans. Therefore, more human studies need to be conducted to show the extent of its utility.

As a final analysis, the fact that PDT offers a minimally invasive approach, has an exceedingly broad spectrum of action against pathogens without posing resistance, causes few reversible adverse effects and that it is already readily available and economic to use suggests that PDT poses a great utility within the field of dentistry. One must be aware of its main limitation, which is the fact that the systemic administration of photosensitizers leads to a local accumulation of them in their target tissue, consequently causing a period of photosensitivity on the skin. Overall, PDT shows great potential as a treatment modality and can be considered an important tool in the treatment of oral diseases, both as the sole therapeutic agent or its use as an adjunctive tool.
